# Monolingual and bilingual language networks in healthy subjects using functional MRI and graph theory

**DOI:** 10.1038/s41598-021-90151-4

**Published:** 2021-05-19

**Authors:** Qiongge Li, Luca Pasquini, Gino Del Ferraro, Madeleine Gene, Kyung K. Peck, Hernán A. Makse, Andrei I. Holodny

**Affiliations:** 1 Levich Institute and Physics Department, City College of New York, New York, NY 10031 USA; 2 Department of Physics, Graduate Center of City University of New York, New York, NY 10016 USA; 3 Department of Radiation Oncology and Molecular Radiation Sciences, Johns Hopkins University School of Medicine, Baltimore, MD 21205 USA; 4 Department of Radiology, Memorial Sloan Kettering Cancer Center, New York, NY 10065 USA; 5 Neuroradiology Unit, NESMOS Department, Sant’Andrea Hospital, La Sapienza University, 00189 Rome, RM Italy; 6 Center for Neural Science, New York University, New York, NY 10003 USA; 7 Department of Medical Physics, Memorial Sloan Kettering Cancer Center, New York, NY 10065 USA; 8 New York University School of Medicine, New York, NY 10016 USA; 9 Department of Neuroscience, Weill Medical College of Cornell University, New York, NY 10065 USA

**Keywords:** Cancer, Neuroscience, Diseases, Medical research, Neurology, Physics

## Abstract

Bilingualism requires control of multiple language systems, and may lead to architectural differences in language networks obtained from clinical fMRI tasks. Emerging connectivity metrics such as *k*-core may capture these differences, highlighting crucial network components based on resiliency. We investigated the influence of bilingualism on clinical fMRI language tasks and characterized bilingual networks using connectivity metrics to provide a patient care benchmark. Sixteen right-handed subjects (mean age 42-years; nine males) without neurological history were included: eight native English-speaking monolinguals and eight native Spanish-speaking (L1) bilinguals with acquired English (L2). All subjects underwent fMRI with gold-standard clinical language tasks. Starting from active clusters on fMRI, we inferred the persistent functional network across subjects and ran centrality measures to characterize differences. Our results demonstrated a persistent network “core” consisting of Broca’s area, the pre-supplementary motor area, and the premotor area. *K*-core analysis showed that Wernicke’s area was engaged by the “core” with weaker connection in L2 than L1.

## Introduction

Human language function is exceptionally complex. Our growing understanding of functional language network (FLN) is enabled by improving techniques to acquire data^1,2^ and using increasingly sophisticated techniques to analyze functional data. This research aims for the latter, and is based on our group’s recently published work in graph theory and *k*-core percolation^3^. We successfully applied this general physics approach to multiple situations, including fMRI analysis of how the brain transition from conscious to subliminal perception^4^, and in the investigation of memory consolidation in rodents^5^. Our model described real and significant findings in multiple scenarios that could not be observed using other methods based on network connectivity alone.

The long-term goal of this research is to use fMRI to understand how language is functionally organized in healthy individuals and how it functionally re-organizes in the brain tumor setting. This understanding may help optimizing and guiding neurosurgical brain tumor resection. This study employs the paradigm selection recommendations recently published in a white paper by the American Society of Functional Neuroradiology^6^. We recommend the use of visually administered, silently generated language tasks that activate language areas related to speech comprehension and production through covert speech, relying on semantic and syntactic mental representations that require word retrieval and articulatory planning^7–10^. The silent word generation task, considered a phonemic fluency task, requires phonologic access, verbal working memory, and lexical search activity, which induce strong activation and lateralization of frontal areas^11–13^. This task showed effective language lateralization in the frontal lobe of the dominant language hemisphere^14^ with optimal language localization^14^ and is considered among the first choices in the state-of-the-art fMRI paradigm for clinical applications^6^.

In a previous study^15^, we established a functional language “core” subnetwork by analyzing 20 healthy subjects without regard to monolingual or multi-lingual status. However, there are known functional differences between the language networks of bilinguals and monolinguals that may affect surgical management^16^. Although the signature of the bilingual language network has been widely investigated, its effect on clinical practice is still unclear. In particular, the common language network of bilinguals in clinically acquired phonemic fluency tasks may differ from that of monolinguals in both the active areas and the connectivity between active nodes. These differences may impact evaluation of the relevance of each language area in clinical practices such as pre-surgical planning^17^.

Language differences are a well-known limitation of fMRI evaluation especially among patients whose native language is different from the language employed in the fMRI task, as the results of the examination may be difficult to interpret. Understanding the interdependence of active fMRI clusters would significantly improve clinical care for minority patients. The ability to identify a specific hierarchy of active clusters on fMRI maps, characterized by a dominant cluster whose integrity is necessary for the stability of the network^4,5^, as well as crucial links between network nodes, is particularly relevant in the bilingual brain, whose peculiar network organization may emerge from clinically-relevant tasks.

The first objective of the current study is to determine how results of clinical fMRI tasks differ between bilingual and monolingual subjects. Second, we study the network architecture of the FLN in each of three groups (monolinguals, bilinguals speaking Spanish, and bilinguals speaking English) and we characterize any difference arising from centrality metrics. Particularly, we sought to assess the *k*-core, which has emerged as an important topological measure of networks because it reveals a robust and highly connected subnetwork, called the *k*-core max (as described in Sec. S1)^3,18^. The *k*-core has previously been employed to measure the stability of the most resilient functional structures in the brain^4,15^ and may provide useful insights in addition to the functional connectivity map. To this end, we analyzed fMRI scans from 16 healthy subjects: eight bilinguals and eight monolinguals. We hypothesized that the k-core method of analyzing fMRI data would demonstrate differences in language organization in the three groups that would not be discernible by standard methods^19–21^.

## Materials and methods

All our methods were carried out in accordance with relevant guidelines and regulations.

### Subjects

We recruited 16 healthy (no neurological history) self-reportedly right-handed adult volunteers (mean age = 42.37 years; standard deviation (SD) = 8.92; nine males and seven females). As our ultimate goal is to apply the current methodology to patients with gliomas involving language areas, and acknowledging that language is organized differently across various age groups, we chose our volunteers to reflect the age distribution of the patients that we will be studying in the future.

Informed consent was obtained from all subjects. The 16 subjects included eight monolinguals (speaking only English) and eight bilinguals (speaking Spanish (L1) as their native language and English (L2) as their second language). All bilinguals had professional-level speaking fluency in English. This study was approved by the Institutional Review Board of Memorial Sloan Kettering Cancer Center (MSKCC).

### Language proficiency tests

Self-reported English and Spanish proficiency data were collected using two independent assessments to each subject by a qualified examiner: the four-item proficiency assessment^22^ and the Language Experience and Proficiency Questionnaire (LEAP-Q)^23^. For both assessments, bilinguals’ English and Spanish proficiency scores were compared using the Wilcoxon signed-rank test (paired). Bilinguals’ English and Spanish proficiency scores were also individually compared to monolinguals’ English proficiency scores using the Mann–Whitney *U* test (unpaired).

This evaluation of the language proficiency was approved by the NIH as part of the grant “Graph theoretical analysis of pre-operative fMRI data in bilingual and English as a second language (ESL) patients with brain tumors.” as part of NIH/NCI U54 CA 137788 (Ahles, PI) CCNY/MSK Partnership for Cancer Research Training and Community Outreach 2013-2019.

### Functional MRI task

All subjects performed a phonemic fluency letter task in response to task instructions delivered visually^6^, as recommended by the American Society of Functional Neuroradiology for the pre-operative planning of brain tumor patients. This choice was motivated by our goal to obtain results directly applicable to the clinical practice. Each monolingual performed the task in English. Each bilingual performed the task in English and Spanish separately, resulting in two separate scans for each bilingual subject. We interchanged the order of English and Spanish tasks randomly. In the final data cohort, we had 24 task-based fMRI (tb-fMRI) scans, eight English scans from the monolingual subjects, and eight English scans plus eight Spanish scans from the bilingual subjects.

In the phonemic fluency task (letter task), subjects were asked to silently generate words that began with a given letter (for example, given the letter “B,” subjects would generate words such as “BIRD,” “BIKE,” “BANK,” etc.). Subjects silently generated words without vocalization to avoid creating artifacts from jaw movement. Stimuli were displayed on a screen over eight stimulation epochs with each epoch lasting 20 s. During the task, two letters were presented in each stimulation epoch. Each epoch also consisted of a 30 s resting period during which subjects were asked to focus on a blinking crosshair. Brain activity and head motion were monitored using Brainwave software (GE, Brainwave RT, Medical Numerics, Germantown, MD. https://www.gehealthcare.com/products/advanced-visualization/all-applications/brainwave) allowing for real-time observation. We specify that the preference of a “covert” task over an “overt” paradigm was motivated by the goal of applying our results in the clinical practice. Less compliance issues and motion artifacts were taken into account to support the choice of a “covert” task. Furthermore, the language paradigm was practiced with each subject prior to the actual fMRI to ensure optimal compliance.

### Data acquisition

Our acquisition process is the same as our previous paper^15^, therefore we repeat this information as follows: A GE 3T scanner (750W, Milwaukee, Wisconsin, USA) and a 24-channel neurovascular head coil were employed to acquire the MR images. Functional images covering the whole brain were acquired using a $T_{2}^{*}$-weighted gradient echo echo-planar imaging sequence (repetition time (*TR*)) divided by (echo time (*TE*)) = 2500 ms/30 ms; slice thickness = 4 mm; matrix = $64\times 64$; FOV = 240 mm; flip angle FA = $80^{\circ }$; voxel resolution = 4 mm $\times$ 4 mm $\times$ 4 mm. In addition, functional coverage matching $T_{1}$-weighted 3D BRAVO (spoiled gradient recalled echo with inversion activated) images (*TR*/*TE* = 8.2 ms /3.1 ms; slice thickness = 1 mm; Inversion Time = 450 ms; matrix = $240\times 240$, FA = $12^{\circ }$) were acquired for co-registration and deformation.

### Data pre-processing

fMRI data were processed and analyzed using the Analysis of Functional NeuroImages (AFNI) software program^24^. Head motion correction was performed using 3D rigid-body registration. Spatial smoothing was applied to improve the signal-to-noise ratio using a Gaussian filter with 4 mm full width of half maximum. Corrections for linear trend and high frequency noise were also applied. Signal changes over time were cross-correlated with a mathematical Gaussian model of the hemodynamic response to neural activation. Cross-correlation involved convolving the modeled waveform corresponding to the task performance block with all pixel time courses on a pixel-by-pixel basis to generate functional activity data. Functional activation maps were generated at a threshold of $p < 0.001$. To reduce false positive activity from large venous structures and head motion, voxels in which SD of the acquired time series exceeded 8% of the mean signal intensity were set to zero. We then addressed multiple comparison correction by performing a cluster correction analysis. All clusters made of contiguous voxels that exceeded the family-wise error rate of $P = 0.05$ were disregarded.

### Individual brain network construction

**Figure 1:**
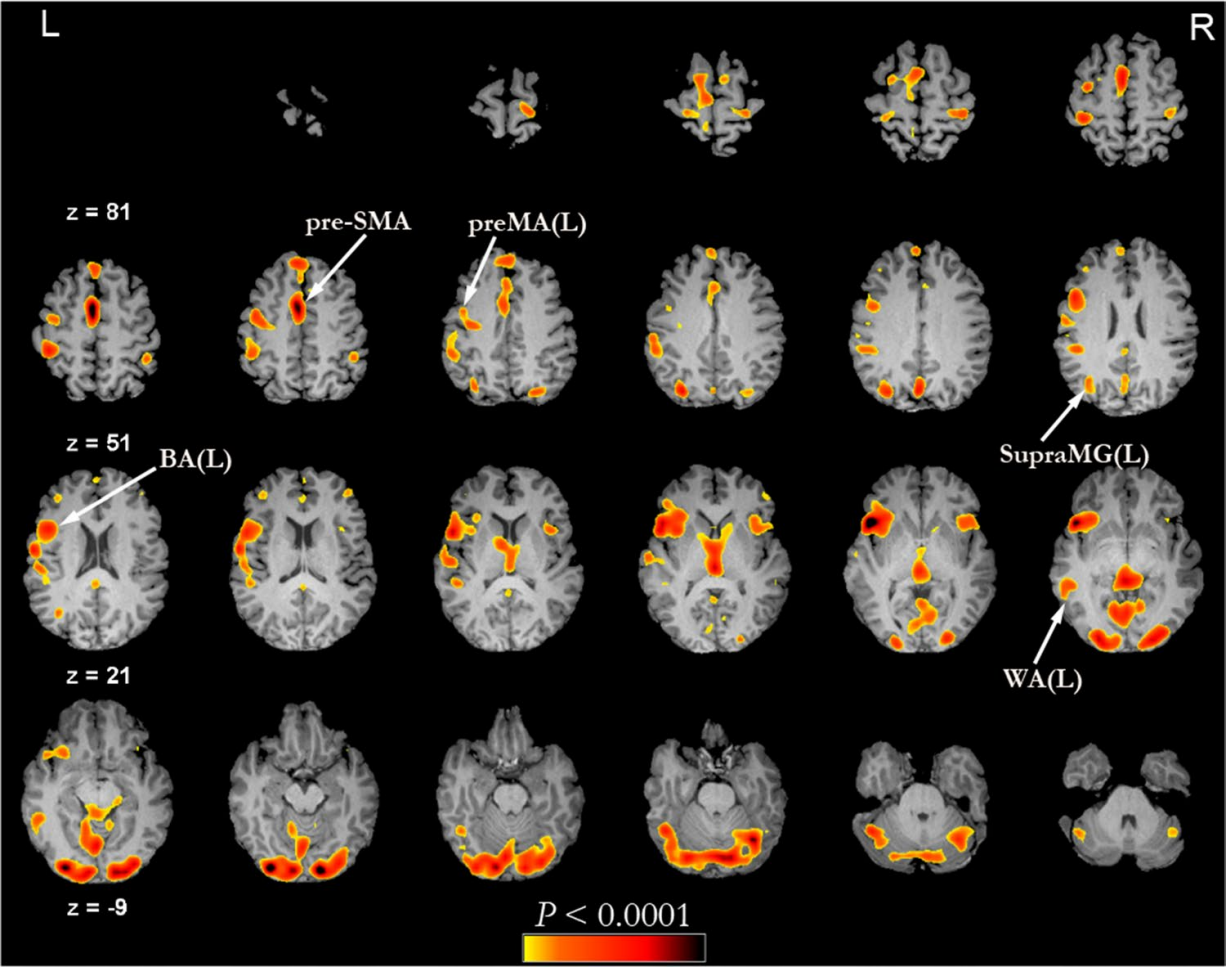
A representative subject’s fMRI activation map overlaid on the anatomical MR image. The reader’s left-hand side is the subject’s left brain hemisphere. The slice number is indicated by z. The areas highlighted in color correspond to fMRI active brain areas and the color bar at the bottom of the figure provide the *p*-values. Several regions have been labeled according to anatomical location. Areas such as visual cortex, which are unrelated to language but active during the fMRI task, were not included. 3D Clusters were extracted and named according to their anatomical locations, such as left Broca’s Area (BA(L)), left Wernicke’s Area (WA(L)), pre-supplementary motor area (pre-SMA), left premotor area (preMA(L)) and left Supra-Marginal Gyrus (SupraMG(L)), as marked on the image.

Following a previously published approach^5,15^, we briefly illustrate the construction method of the functional network on two different scales: voxel and fROI. At both scales, the network construction starts from the identification of the fMRI active voxels under the task performance described in “Functional MRI task” section. Active voxels were identified as those which passed a statistical significance tests corrected for multiple comparison, as described in “Data pre-processing” section. A sample subject’s resulting fMRI activation map is shown in Fig. 1. Since instructions were delivered visually, the visual cortex inevitably activated. As functional activation in the occipital lobes was a consequence of the paradigm delivery rather than the subsequent language processing, these areas were discarded from the analysis.

At the **voxel scale**, each active voxel is defined as a node in the functional network. Active voxels that are contiguous and in the same anatomical regions are labeled as belonging to the same functional region of interest (fROI). The labeling of the fROIs based on their anatomical location was performed by a neuroradiologist with 20 years’ experience in clinical and research fMRI^7,8,25–28^. Since there exist high inter-subject variability in the mapping of cognitive functions, we did not determine fROIs at the group level but rather at the individual level^29^. It is worth noticing that, due to the presence of mass effect in brain tumor patients, pre-operative fMRI results are always interpreted on an individual level^30^.

**Figure 2:**
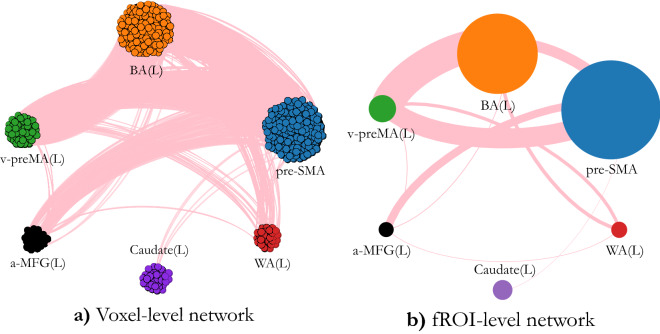
A representative bilingual subject’s network on the voxel scale (**a**) and fROI level (**b**). In (**b**) each node represents an fROI, and the node’s size is proportional to the number of voxels in the fROI. The thickness of each link connecting two fROIs is proportional to the sum of all link weights inter-connecting the voxels between the two fROIs (as in Eq. (2)). Visualization of (**a**) was achieved by using Gephi^32^ and visualization of (**b**) was partially achieved by python 3.7.

Next, we defined the links between voxels (nodes) by following standard methods of measuring statistical dependencies between activated voxels^5,15,19–21^. Links between voxels were obtained by thresholding the pair-wise voxel correlation of the Blood-Oxygen-Level Dependent (BOLD) signal^15^:

1 $$\begin{aligned} C_{ij}=\frac{\langle x_i x_j \rangle - \langle x_i \rangle \langle x_j \rangle }{ \sqrt{(\langle x_i^2 \rangle -\langle x_i \rangle )^2 (\langle x_j^2 \rangle -\langle x_j \rangle )^2 } }. \end{aligned}$$

where $x_i$ is a vector encoding the fMRI time response of voxel *i* and $\langle \cdot \rangle$ indicates a temporal average. If a pair of voxels has correlation absolute value above a given threshold then the pair is considered connected by a link, otherwise the two voxels are not linked. In Fig. 2a, we display a realization of the voxel scale network for the sample subject of Fig. 1. Each node represents a voxel, and nodes belonging to the same fROI are colored equally. Links connecting a pair of voxels belonging to different fROIs are shown as pink lines. The links connecting pairs of voxels within the same fROI are not displayed.

At the **fROI scale**, each node in the network is simply an entire fROI which contains all the active voxels belonging to the same anatomical area. To define the connectivity at the fROI scale, the functional link weight ($W_{ij}$) between two fROIs (labeled *i* and *j*) is defined as the sum of all the binarized functional link weights ($w_{lm}$) connecting all pairs of voxels (*l*, and *m*) between the two fROIs, normalized by the sum of the two fROI sizes ($S_i$ and $S_j$):

2 $$\begin{aligned} W_{ij} = \frac{\sum _{l,m \in \{i \leftrightarrow j\}} w_{l,m}}{ (S_{i}+S_{j})} \end{aligned}$$

Thus, there exist a nonzero fROI-fROI connection between any pair of regions such that a single voxel in each region is inter-connected. We show a realization of an fROI-level network of the same representative subject in Fig. 2b. Here, each colored node represents a fROI. The thickness of each link connecting two fROIs is proportional to the link weight (*W*). The the fROI-scale link’s thickness connecting two nodes in Fig. 2b, may appear inconsistent with the amount of links connecting the two same fROIs in the voxel-scale network (Fig. 2a). This visual difference is due to the normalization present in the normalization factor in Eq. (2).

At this point, an important clarification about the interpretation of the voxel- and fROI-scale network is necessary. To construct the functional network we follow a two steps procedure: (i) we select the fMRI active voxels during the task, (ii) we compute the pair-wise correlations among the active voxels to determine the network’s links. With this procedure, regions that are highly active in a block design task are also highly correlated across time due to the task. This means that, voxels which are highly correlated with the task stimulus are also very likely to be correlated with each other. If the purpose of the analysis here was to determine the connectivity of the language network *independently* from the task, this procedure would cause a *circularity* issue, also know as *double dipping* in the literature, as very well reported in^31^. When the same data is used for selection and selective analysis, indeed, the descriptive statistics is distorted due to the presence of the noice in the data^31^. In our approach here, we first select voxels that are active during the task and then we investigate which ones are more correlated among themself *given that* they are also active during the task. In other words, our results are task-*conditioned* or task-*based*. This means that the resulting functional language network is not an independent architecture from the task but, on the contrary, it depends on both the task and the selective analysis. We stress that this approach is rather different from standard resting state approaches to construct functional networks, where the selection is usually done on the only anatomical basis, as discussed in^31^. Here, we are interested in highlighting a language network that is task-dependent, or task-conditioned, and we further want to analyze its properties. As a consequence, all our results will be dependent on the voxel selection, i.e. the initial activation map. This is fine as long as we keep in mind that the resulting language network is task-conditioned and, therefore, specific to the particular task that we use in our experiment.

We constructed both voxel scale and fROI scale networks from the tb-fMRI signal for each of the 24 individual scans of data partitioned into three groups: monolinguals, bilinguals speaking English, and bilinguals speaking Spanish, with each group containing eight networks. Next, we measured the common network characteristics at the group level to estimate robust connectivity across subjects within a group.

### Common network construction across subjects

We named the persistent functional architecture across subjects in a particular group at the fROI-level the “*common network*”. This common architecture was constructed for each group by retaining only those pairs of fROIs and those functional links connecting them that were present across all subjects, and we considered the number of appearances of the functional link within the group as a measure of frequency.

The weight of the functional link connecting two fROIs (*i* and *j*), in the common network ($W_{ij}^{\mathrm{C}}$) was defined as the average of the ${W}_{ij}$ connecting those fROIs across subjects:

3 $$\begin{aligned} W_{ij}^{\mathrm{C}} = \frac{1}{N} \sum _{l=1}^N W_{ij}^{(l)} \end{aligned}$$

where *N* is the number of individuals and where the link $W_{i,j}$ is nonzero.

## Results

### Language proficiency tests

From the Mann–Whitney *U* test (unpaired), no significant differences were found between bilinguals’ English proficiency and Spanish proficiency in any language domain (speaking, understanding, reading) or in overall proficiency ($p > 0.05$). There were also no significant differences between monolinguals’ English proficiency and bilinguals’ English proficiency across all measures ($p > 0.05$). There were no significant differences between monolingual’s English proficiency and bilinguals’ Spanish proficiency ($p > 0.05$). There were no significant differences in self-reported English and Spanish proficiency for monolingual and bilingual participants.

### Individual networks

From the 24 brain scans, 17 activated areas (or fROIs) were identified by the procedure described above (2.2.6). A summary of these activated areas and their frequency of activation by subject is shown in Supplementary Table S1. Both hemispheres demonstrated activation; however, left hemisphere dominance is clearly observed, which is expected in an fMRI language task among right-handed subjects, as language brain activation is mostly concentrated in the left hemisphere in right-handed individuals^33^.

Although 17 activated areas were detected, not all areas were activated in each subject due to inter-subject variability. We observed that the most areas were activated in the monolingual group (16/17), followed by the bilingual Spanish-speaking group (13/17) and the bilingual English-speaking group (12/17).

The areas that were activated in all subjects and all groups were the pre-supplementary motor area (pre-SMA), Broca’s Area (BA(L)), and ventral premotor area (v-pre-MA(L)). Wernicke’s Area (WA(L)) was activated in all eight subjects of bilingual Spanish-speaking group. Thus, these regions were included in the corresponding common networks by default. The anterior Middle Frontal Gyrus (L) (a-MFG(L)) and the Supra-Marginal Gyrus(L) (SupraMG(L)) activated with significant frequency (50 to 75 percent of subjects) in all three groups.

Individual link weights for the fROI scale network are reported in Supplementary Tables S2–S4. We observe that, overall, the preMA is the most connected area across subjects in terms of connectivity weight (strength). Only the shared links between subsets of activated fROIs are shown in these tables, with the subsets representing the core similarities between the groups included in the common networks. As a general trend, the strongest link (with the largest connectivity weight) is between v-preMA(L) and BA(L), followed by the link between v-preMA(L) and pre-SMA, and then the link between pre-SMA and BA(L).

We note several relatively small fROI-level link weights such as $W_{ij} =0.01$ in some subjects (for example, refer to the connection between BA and WA for Subject 6 in Supplementary Table S2. These small values arise because the fROI scale network normalizes the link weights by fROI size (number of voxels in the clusters), which can lead to apparently small link weights in some cases when fROI size is large compared to relatively sparse interconnections.

### Common networks

**Figure 3:**
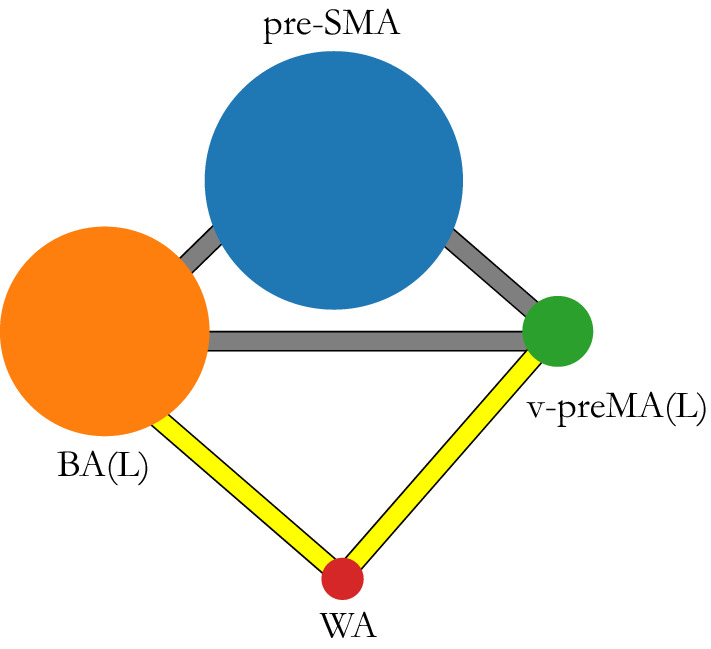
Common network structure in fROI level. The colored nodes represent fROIs, and their size is proportional to the averaged size of fROIs across all subjects. The solid gray links connecting pre-SMA, BA(L), and v-preMA(L), is the “$\bigtriangleup$ structure,” and the yellow links connecting WA(L) to BA(L) and to v-preMA(L), respectively, is the “$\bigvee$ structure”. We use different colors for the links to distinguish their different frequencies ($f_{i}$) of activation (activate in # of subjects/the total # of subjects). This information is provided as in Table 1: the “$\bigtriangleup$ structure” was activated in all studied subjects. The “$\bigvee$ structure” was activated in all subjects in the bilingual Spanish-speaking group; this is different from monolingual group and bilingual English-speaking group, which only activated 75% and 50% of the time, respectively.

The resulting common networks were constructed as described in “Common network construction across subjects” section. In each group, the shared common network contained only nodes and links that were present across the majority of subjects. A visualization of the shared common networks at the fROI level are shown in Fig. 3. All groups’ common networks have a similar fully connected structure involving the pre-SMA, BA(L), and v-preMA(L) (also called the “triangle structure,” notated by $\bigtriangleup$) across all studied scans (n = 8/8 in each group; 100%). This structure is identified by the consistent edges connecting fROIs across individuals. Therefore, this triangle captures the part of the individual functional network that goes beyond inter-subject variability, i.e. the part of the individual network that is common across individuals. This structure is consistent with the results of our recent study of healthy individuals performing a different clinical pre-operative language task, in which it was called the “core” of the language network^15^.

Another structure involving WA(L)–v-preMA(L) and WA(L)–BA(L) (called the “V structure,” notated as $\bigvee$) is present in 6/8 (75%) subjects in the monolingual group, 4/8 (50%) subjects in the bilingual English-speaking group and 8/8 (100%) subjects in the bilingual Spanish-speaking group. This information is also summarized in Table 1. These four regions are functionally connected with one another, with detailed modular link weights shown in Supplementary Tables S5–S7.

**Figure 4:**
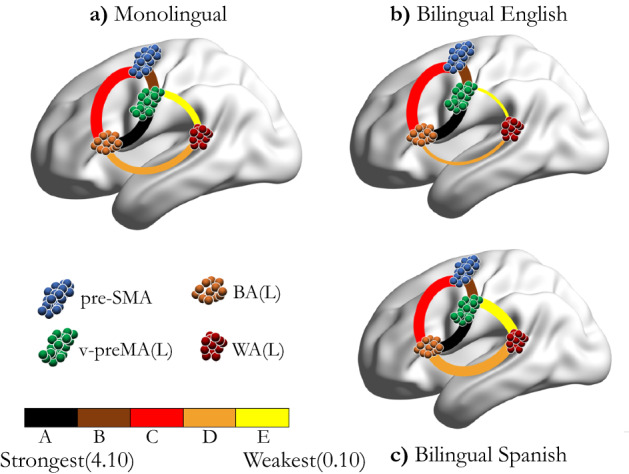
Common network visualization. Visualization of the shared common network across subjects in (**a**) monolingual group, (**b**) bilingual English-speaking group and in (**c**) bilingual Spanish-speaking group, constructed by the methods described in “Common network construction across subjects” section. Here, we show the sagittal view of the left brain. The modules color legend is provided below panel (**a**). The link colors represent the $W_{ij}^{\mathrm{C}}$ hierarchy strengths within each group. The link color bar is provided below the color legends of the modules. From Left to Right, we show the strongest (the largest $W_{ij}^{\mathrm{C}}$) to the weakest (the smallest $W_{ij}^{\mathrm{C}}$). The links between each fROI pairs are abbreviated as A-E, see Supplementary Tables S5–S7 for more details. The thickness of the links represents how frequently (in how many subjects) they appear. WA connects with preMA(L) and BA(L) in 75% of monolingual subjects, 50% of bilingual English-speaking subjects and 100% of bilingual Spanish-speaking subjects, respectively. The background brain surface was created in BrainNet Viewer^34^.

Although sample SD in the link weights for each group is large relative to the mean, it is nevertheless evident that the common modular link weights are consistently larger for bilingual subjects speaking their native language (L1) than for bilinguals speaking their second language (L2), as shown in Supplementary Tables S5–S7. A larger modular level link weight stems from the higher density in inter-fROI voxel level connections between common fROIs. The larger modular link weight in the bilingual Spanish group coincides with a 100% attachment level of the $\bigvee$ structure. The average common link weights of monolinguals tend to lie between those of bilingual Spanish-speaking and bilingual English-speaking subjects. The relative common modular link weights as determined by Eq. (3) are indicated by the color bar in Fig. 4. The thickness indicates the in group occurrence frequency $f_i$ of the link.

**Table 1:** Frequency ($f_i$) of each structure appearance.

	Monolingual subjects	Bilingual subjects	
	English task	English task	Spanish task
$\bigtriangleup$ structure	8/8 ($100\%$)	8/8 ($100\%$)	8/8 ($100\%$)
$\bigvee$ structure	6/8 ($75\%$)	4/8 ($50\%$)	8/8 ($100\%$)

The shared common networks reveal a hierarchical ordering of link weight as shown in Fig. 4. We denote the link BA(L)-v-preMA(L) by A, pre-SMA-v-preMA(L) by B, pre-SMA-BA(L) by C, BA(L)-WA(L) by D and WA(L)-v-preMA(L) by E. The link weight hierarchy ($A> B> C> D > E$) is consistent in all three groups. These results are consistent with the findings in our previous investigation conducted with 20 right-handed healthy controls^15^. In the previous study^15^, we named the four fROIs the language “core” sub-structure for the specific language task under study.

Although we have grouped the bilingual English and bilingual Spanish common networks separately, they are constructed from the same group of eight subjects performing the task in different languages. The monolingual group represents a different set of individuals. Therefore we expect an extra factor of inter-subject variability when making monolingual to bilingual group level comparisons as opposed to L1 to L2 based comparisons within the bilingual group.

Note that not all pairs of core modules were directly connected. This is to be expected since, for example, pre-SMA and WA(L) have no known structural connections, whereas the WA and BA are known to be connected by the arcuate fasciculus^35^. The absence of a link does not convey direct information about the underlying structural connectivity due to intra- and inter-subject variability in subject response to the task paradigm as noted above.

Our primary findings are the differential attachment of the $\bigvee$ structure between groups and the higher common link weights in the L2 group compared to the L1 in the $\bigtriangleup$ structure.

### *K*-core analysis

The *k*-core of a given architecture is defined as the maximal sub-graph (not necessarily one that is globally connected) of all nodes having a degree (number of connections) of at least *k*. To partition the whole network into hierarchically ordered sub-networks, we iteratively prune all nodes with degree *k* until further pruning is no longer possible (when pruning has caused the whole network to collapse completely)^36^. The removed nodes are in the *k*-shell and the remaining subnetwork is called the $(k+1)$-core. This final step will lead to finding the nodes in the maximum shell and the most connected sub-graph (maximum core) just before the whole network collapses. This process is called *k*-shell decomposition. We provide a brief explanation of the *k*-core, the *k*-shell decomposition algorithm, and the meaning of the $k_{max}$ and *k*-shell occupancy histograms through a schematic *k*-shell decomposition process in Supplementary Fig. S1.

It has been shown that for networks with positive couplings (positive link weights), the *k* max core is the component of the system that is most resilient with respect to network failures, where in this case a failure means a reduction of the link weight (potentially due to brain tumor invasion or physical resection)^3^. All thresholded voxel-voxel BOLD time series correlations defining the link weight for our experimental task paradigm were positive. Thus, conducting *k*-core analysis would reveal the most robust component of the functional language architecture in healthy monolingual and bilingual subjects.

**Figure 5:**
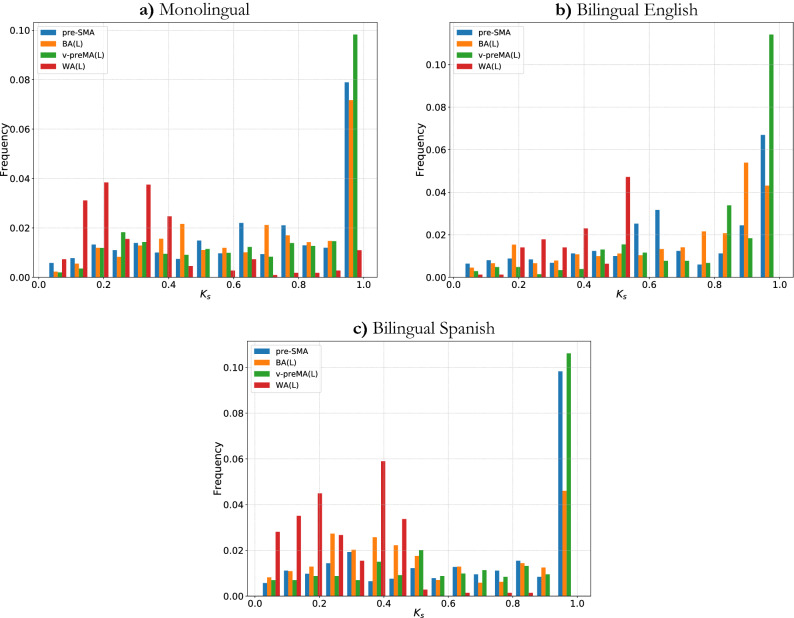
*K*-shell occupancy for all three groups. Different colors represent nodes belonging to different modules, and the color legend is shown in the upper left of the figure. In (**a**)–(**c**) pre-SMA, BA(L), and v-preMA(L) peak at the maximum shell; however, WA(L) occupies most of the middle and low shell. In (**b**), WA(L) does not occupy the higher ($k_{s}$ > 0.5) shell at all.

The *k*-shell (occupancy) histogram provides important and direct insights into the network structures. Therefore, for each group, we calculated the $k_s$ shell occupancy for each node in the individual network *at the voxel level*. Then, we normalized $k_{s}$ by the maximum shell number found in each individual network ($k_{max}$) so that $k_{s}$ ranged from 0 to 1, where $k_{s}$ = 1 is the maximum shell (*k* max core). Next, we collected the individual networks’ nodes together, regardless of which subject they came from, and placed them into 15 bins according to their $k_{s}$ values. Then, we grouped the nodes in each bin by the modules to which they belonged. Finally, we plotted one unique *k*-shell occupancy histogram for each group. The histograms are shown in Fig. 5 for the four modules from the common shared core of the FLN: pre-SMA, v-preMA(L), BA(L), and WA(L). Panels a–c display the *k*-shell histogram for monolinguals, bilinguals speaking English, and bilinguals speaking Spanish, respectively.

**Figure 6:**
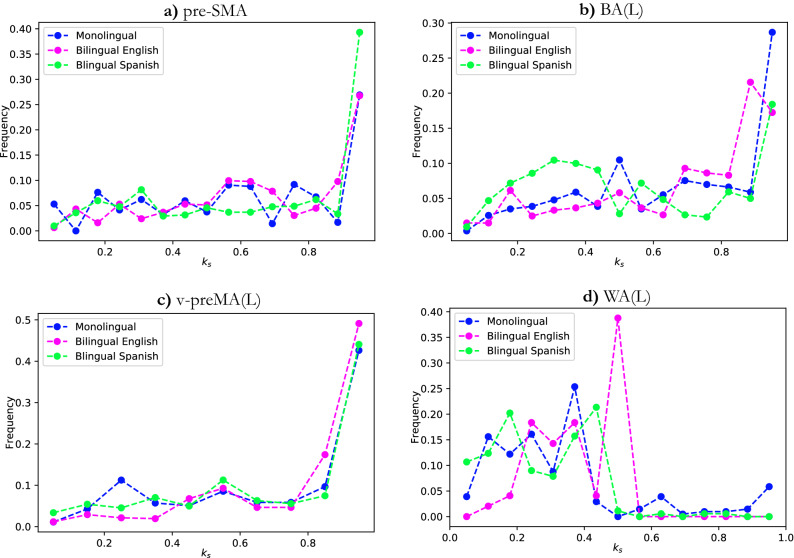
(**a**) Pre-SMA, (**b**) BA(L), (**c**) v-preMA and (**d**) WA *k*-shell occupancy for all three groups. Different colors represent different groups. Notice that the three colored curves are most distinctive in (**d**). For this panel only, the most distinctively behaving group is bilingual English-speaking group, but all three groups appear to behave quite differently.

In parallel, we plot each module’s *k*-shell histogram separately, as shown in Fig. 6. To validate our results, we conducted residual analysis to determine how much differences in *k*-shell occupancy distributions between groups. To this end, we compute the sum of squared errors (SSE or residuals) between each distribution pair for each of the four modules. By the sum of squared errors, we mean that the differences between data points from different distributions in the same bin and then for each distribution pair, we sum all such squared differences.

**Figure 7:**
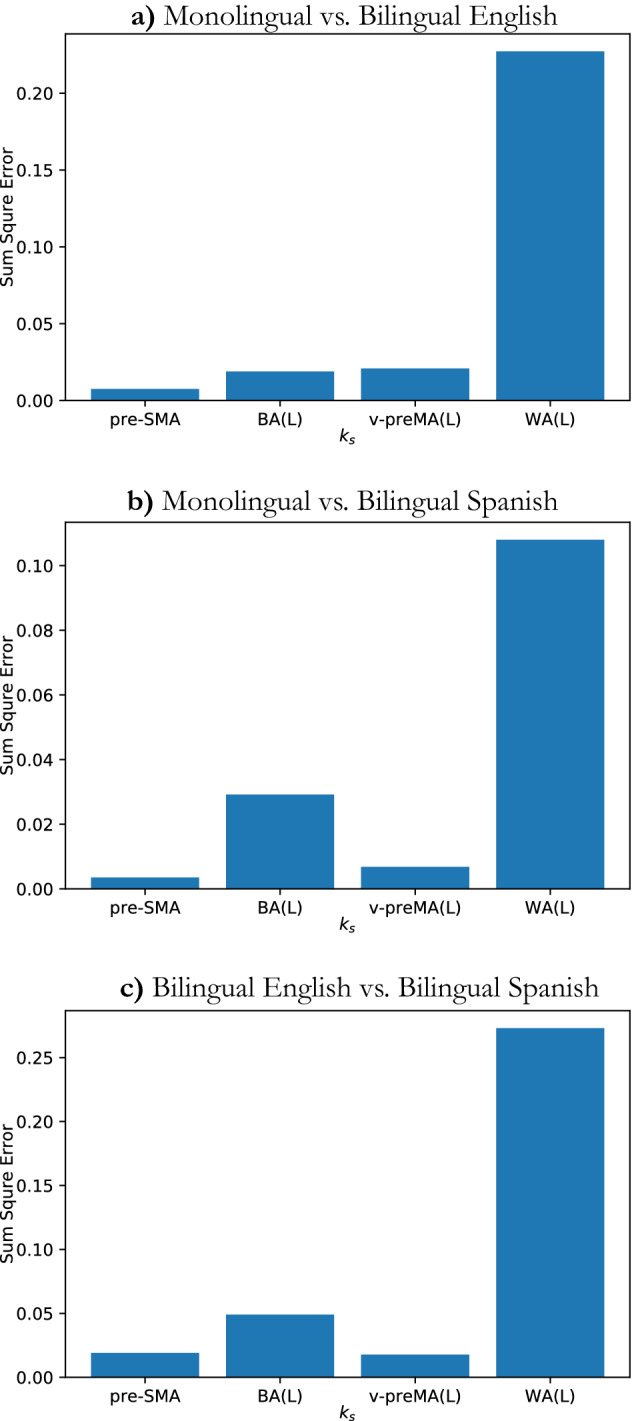
Sum of square errors (SSE) of *k*-shell occupancy for each module. For all three pair of groups, as shown in (**a**) to (**c**) WA(L) distribution had the largest SSE compared to the “core” modules. Bilingual English and Spanish speakers’ SSE in WA, as seen in (**c**), has the largest value, while monolingual and bilingual Spanish-speakers have the smallest SSE in WA.

First, we observed that the shell occupancy of the “core” modules - BA(L), v-preMA(L), and pre-SMA were quite similar in all three groups; most nodes in these three modules occupy the maximum shell ($k_{max}$). This can be seen in Figs. 5a–c and 6a–c. This is also confirmed by residual analysis (Fig. 7). By similarity in distribution, we mean that the SSE is relatively small in the core modules compared to the WA(L), which displays much greater sums of square residuals.

Second, the occupancy of WA(L) is fundamentally different from that of the “core” regions, as most nodes do not occupy the maximum shell. Rather, occupancy is distributed among the smaller shells ($k_{s} \le 0.5$) in the peripheral of the network. This is observed consistently (in Fig. 5a–c as well as Fig. 6d) among all three groups: monolingual, bilingual English-speaking and bilingual Spanish-speaking. This is supported by SSE analysis (Fig. 7a–c), which shows that WA(L) is five to ten times greater in the SSE than in the “core” modules. Notably, the above two points align with recent findings that WA(L) may belong to the lower shells^15^ rather than the “core” regions.

Furthermore, comparing the groups for WA(L) occupancy, important and statistically significant differences between the three groups emerged. For example, as shown in Fig. 6d, the occupancy of WA(L) in the bilingual English-speaking group peaked at $k_{s} = 0.5$ and did not extend to higher shells. This result suggests that the WA may be less resilient in its attachment to the FLN in bilingual subjects speaking English (L2). Additionally, in the bilingual English-speaking group, WA(L) shows less occupancy in the peripheral outer shells (small $k_{s}$) than in the other two groups. Visually, the occupancy distribution of WA(L) in monolingual and bilingual Spanish-speakers appeared to be more similar to one another than to the bilingual English-speaking group. These apparent difference are corroborated by the SSE analysis shown in Fig. 7a–c.

To summarize the results of the SSE analysis, the “core” *k*-shell distribution tended to be similar between groups, whereas for the WA, the *k*-shell histogram varied significantly between each group pair. Furthermore, we found a larger SSE ($\sim$ 0.2–0.25) in the occupancy of WA when we compared the bilingual English-speaking group to either the monolingual group or the bilingual Spanish-speaking group, as shown in Fig. 7a,c. On the other hand, the SSE of WA drops to less than half of this value (SSE $\sim$ 0.1) when we compare the monolingual with the bilingual Spanish-speaking group, implying that the occupancy of WA is more similar between the monolingual and the bilingual Spanish-speaking group than between the monolingual and the bilingual English-speaking group.

Residual analysis suggests that the occupation behavior of WA is unlikely to be caused by random noise in the data, and more likely reflects real underlying trends.

## Discussion

The goal of this study was to identify the hierarchal relationship of language areas identified by fMRI in monolingual and bilingual subjects with the eventual goal of applying these results to the brain tumor neurosurgical setting. We used network analysis to study the structure and interconnections between essential language areas in both monolingual and bilingual groups. We conducted this study by first constructing FLNs on the individual level and aggregating these results by group to identify group structures and to highlight differences between groups.

For the common networks, we observed that there were both similarities and differences between the FLN of monolinguals and bilinguals. All groups share a core network composed of a resilient “triangular structure.” The “triangular structure” also connects to WA to form the “V structure” with different degrees at the group level: 8/8 bilingual subjects speaking Spanish, 6/8 monolingual subjects, and 4/8 bilingual subjects speaking English. Bilingual English-speaking subjects displayed the smallest common link weights, while the same subjects performing the fMRI task in Spanish displayed the largest common link weights. These results reflect the higher clinical task engagement of L1 language processing systems when compared to L2 systems.

The hierarchy of strengths between the three clusters ($A> B> C> D > E$) is consistent across all three groups, as shown in Fig. 4 and Supplementary Tables S5–S7. These findings are consistent with our previous analysis of 20 right-handed healthy subjects^15^. This consistent hierarchy may predict the amount of information traffic flowing between each interacting module. The low connectivity weight between ventral preMA and WA (see Supplementary Tables S5–S7) may be explained by the increased distance between the two structures.

One of the results of our study may at first appear somewhat puzzling: the apparently great role in language organization played by the preMA (L) compared to one of the classical language areas: WA (L). The answer is that in the present study, we are not evaluating language in general (if such a thing is even possible), but are considering a specific paradigm, designed to analyze a specific problem. The paradigm that we employed is known to highlight the frontal language areas (BA) rather than the temporal language areas (WA). Additionally, we delivered the paradigm visually. This means that the information traveled as follows: visual cortex (occipital lobe) $\rightarrow$ frontal eye fields (posterior aspect of the middle frontal gyrus, or preMA(L)) $\rightarrow$ Exner’s area (posterior aspect of the middle frontal gyrus, or preMA(L)) $\rightarrow$ the language network (including Broca’s, Wernicke’s, pre-SMA and the anterior aspect of the MFG, which houses verbal working memory).

Exner’s area coordinates visual information from the occipital lobe (primary visual cortex) and the frontal eye fields with the language network^27^, and is critically involved in transforming phonological representations of words into motor commands for handwriting^37^. Therefore, although we eliminated the activation in the occipital lobes (the primary visual cortex), a consequence of the paradigm design was the heightened activation of the preMA(L). Had we delivered the same paradigms aurally, we may have seen activation of the language network plus the auditory network that leads up to the language network. This activation would likely have included Heschl’s gyrus (the primary auditory network) but not the visual cortex, frontal eye fields and Exner’s area. This difference has, in fact, been our clinical experience with brain tumor patients when we moved from an aural presentation fMRI paradigm to a visual paradigm.

Nevertheless, our results confirm the important role played by the left ventral preMA in language processing, regardless of which language the subject speaks. The premotor cortex is known to participate in bilingualism through the dorsolateral prefrontal cortex (DLPFC). Nevertheless, its participation in the core and its relationships with other components is incompletely understood. As mentioned in “Introduction” section, the premotor area should control language selection. In our results, the consistent activation of preMA can be partly explained by its role in the extended BA, as discussed in our previous work^15^, which may be valid regardless of the language spoken. Particularly, the strong link between the opercular BA and the ventral premotor cortex should constitute a hub for language production by connecting with the SMA^13^.

Increasing evidence pointed to a more comprehensive role of the cerebellum in language processing besides motor coordination^38^. This is also true in pathologic conditions. For example, cerebellar activations showed interesting reorganization phenomena in the setting of brain tumors^39^, which may help predicting the behavior of main language areas. However, a consensus regarding the exact role of the human cerebellum in linguistic function is still missing^38^. In this study, we wanted to investigate core-components of the language network of bilingual versus monolingual subjects. As a consequence, most of secondary language areas did not appear in the *k*-core, although they may display distinctive features in bilinguals. We believe that different approaches are needed to investigate interdependency of primary and secondary language areas in detail, including cerebellar activations.

Although the correspondence between structural and functional connectivity is not yet fully understood^19,40^, our results may be supported by structural evidence such as known white matter bundles connecting the network core nodes such as the frontal aslant tract and the dual pathways of language^15^.

As a minor difference between the groups, we found more frequent activation of secondary language areas in bilinguals. This result aligns with previous studies that demonstrated that the left Caudate and Angular Gyrus are relatively more involved with bilingualism^41,42^.

With respect to the *k*-core analysis, we see in Fig. 5 that the WA in the bilingual English-speaking group does not populate the lower *k* shells, and that WA populates more in the lower *k* shells of the bilingual Spanish-speaking group than in the monolingual group. Monolinguals populate the *k*-max core, whereas the bilingual groups do not. WA populates the *k*-max core less than in the core, but still significantly.

The *k*-core max must be highly connected to other highly connected nodes, whereas the low shells resemble dangling ends of the system. In general, the *k*-shell represents a hierarchy of nodes. Bilingualism manifests itself in a reduction of the most important nodes, at least as far as WA is concerned. These results cannot be obtained with methods based only on the activity of the brain, and network analysis is necessary to differentiate the three states of the WA. Our network analysis captured differences in the role of WA usually unnoticed in conventional task-based fMRI evaluation.

We consistently observed that the greatest portion of the three modules pre-SMA, BA(L), and v-preMA(L) occupied the largest, *k*-max shell. This suggests that the triangle’s modules are the most resilient part of the network, which prevents cascading failures in the event of network failures^3,18,43^. This sub-structure may thus prevent network collapse in the event of removal of links caused by pathological conditions and/or subsequent surgical intervention. In either case, damage to these core links may result in irreversible damage to the language network.

It should be noted that the presence of WA in the weaker *k* cores does not necessarily imply that WA is less important to the language network. Rather, weakly connected nodes sometimes play pivotal roles in the network processes. Morone et al.^44^ have shown that nodes with low degrees are sometimes the most important essential nodes when they hold the keys to connections between hubs (modules). In this context, the *k*-core results may indicate that WA(L) has at least a distinctive functional nature to the other three core members of the FLN, with respect to the network path structure. Recent studies evidenced distinct anatomical substrates for the motor-speech and lexico-semantic systems, suggesting a double triangular network serving lexico-semantic processes and speech articulation^13^. The areas wired in this network show an intriguing correspondence with our results and structural evidence from the literature^45^.

The results of our *k*-core analysis may also be supported by structural evidence. We found a significant difference in the *k*-shell distribution of WA(L) nodes between the L1 and L2 tasks. The other common fROIs occupied the maximum shell with different proportions in L1 versus L2 as shown in Fig. 5. In fact, there is evidence for significant differences in structural connections between monolinguals and bilinguals. Diffusion tensor imaging (DTI) studies have demonstrated that the representation of uncinate fasciculus, which connects the deep opercular cortex with the superior anterior temporal lobe^46^ is increased in bilinguals^16,47^. Similarly, increased fractional anisotropy of the superior longitudinal fascicle, connecting the preMA(L) with the superior temporal gyrus, has been reported in bilinguals^16,47^. These results may support an individualized approach regarding task choice for clinical fMRI in bilingual patients: administration of language tasks in L1 instead of L2, or both L1 and L2, may provide more information about the bilingual network. This consideration is based on the higher task engagement of L1 language processing systems, as evidenced by the higher common network link weights and more frequent involvement of the “V-structure”.

### Limitations

A potential limitation of this study is the sample size, which may affect the statistical significance of network properties due to inter- and intra- subject variability. Further studies are needed to confirm our results in larger populations and to provide additional evidence to support our hypotheses. In this study, we evaluated only one language paradigm. Although the selected task is highly relevant for clinical practice, as confirmed by the literature and our own experience, valuable information is certainly contained in other language tasks, which should be investigated in future studies. It is important to mention that language network organization changes substantially with age, especially for challenging tasks like phonological word generation. Our group-age was limited to adult patients, where lateralization is considered less variable^48^, and we deem our age group homogeneous enough to prevent significant age-related biases. We stress that future analysis on the matter should include more subjects from a wider age range and consider chronological age as an additional factor in the analysis.

Few limitations affected the interpretation of cerebellar activity in our study. This included exuberant visual/occipital activation and/or venous flow artifacts from transverse sinuses masking cerebellar activation. As pointed out above, future researches with different approaches will unravel the interdependency of primary and secondary language areas in detail, including cerebellar activations.

This study investigated the language network connectivity from the fMRI paradigms suggested as clinical reference by the ASFNR^49^, which are covert tasks. However, covert versions of word generation paradigms are not exempt from limitations, including difficulties in response monitoring and activation of extralinguistic functional components (related to divided attention, response conflict, and inhibition) which may affect the linguistic interpretation of regional activations^50^. Paced overt fluency paradigms with sparse/clustered acquisition showed promising results in clinical contexts^51,52^ and may represent a valuable alternative to covert paradigms. The application of our method on overt tasks may further improve our understanding of language areas interdependency, representing an avenue for future research.

Finally, although language skills were evaluated through the LEAP-Q and proficiency questionnaires, a formal and more extensive neuropsychological evaluation could provide further information regarding subject performance. This should be taken into account in future studies.

## Summary

Both monolingual and bilingual subjects share a common language network formed by BA, preMA, and pre-SMA that occupies the $k_{max}$ shell and shows features of a central core for language across groups, consistent with our previous results on healthy subjects^15^. Moreover, WA is engaged by the network core with variable extent across groups (8/8 bilingual Spanish-speaking subjects, 6/8 monolingual subjects, and 4/8 bilingual English-speaking subjects), reflecting different *k*-core occupancies. The major difference in groups is that the bilingual L2 group’s nodes occupied only the lower half of the *k* shells. The bilingual L2 group also showed weaker connection strengths between core fROIs compared to when the same subjects performed the task in L1.

## Supplementary Information

Supplementary Information 1.

Supplementary Information 2.

## Data Availability

This data set is publicly available at: http://www-levich.engr.ccny.cuny.edu/webpage/hmakse/brain/.

## References

[1] Schluppeck D, Sanchez-Panchuelo R-M, Francis ST (2018). Exploring structure and function of sensory cortex with 7 T MRI. Neuroimage.

[2] Petridou N, Siero JC (2019). Laminar FMRI: What can the time domain tell us?. Neuroimage.

[3] Morone F, Del Ferraro G, Makse HA (2019). The k-core as a predictor of structural collapse in mutualistic ecosystems. Nat. Phys..

[4] Lucini FA, Del Ferraro G, Sigman M, Makse HA (2019). How the brain transitions from conscious to subliminal perception. Neuroscience.

[5] Del Ferraro G, Moreno A, Min B, Morone F, Pérez-Ramírez Ú, Pérez-Cervera L, Parra LC, Holodny A, Canals S, Makse HA (2018). Finding influential nodes for integration in brain networks using optimal percolation theory. Nat. Commun..

[6] Black D, Vachha B, Mian A, Faro S, Maheshwari M, Sair H, Petrella J, Pillai J, Welker K (2017). American society of functional neuroradiology-recommended FMRI paradigm algorithms for presurgical language assessment. AJNR Am. J. Neuroradiol..

[7] Price CJ (2012). A review and synthesis of the first 20 years of pet and FMRI studies of heard speech, spoken language and reading. Neuroimage.

[8] Price CJ (2010). The anatomy of language: A review of 100 FMRI studies published in 2009. Ann. N. Y. Acad. Sci..

[9] Cappa SF (2012). Imaging semantics and syntax. Neuroimage.

[10] Bouchard KE, Mesgarani N, Johnson K, Chang EF (2013). Functional organization of human sensorimotor cortex for speech articulation. Nature.

[11] Li Y, Li P, Yang QX, Eslinger PJ, Sica CT, Karunanayaka P (2017). Lexical-semantic search under different covert verbal fluency tasks: An FMRI study. Front. Behav. Neurosci..

[12] Emch M, von Bastian CC, Koch K (2019). Neural correlates of verbal working memory: An FMRI meta-analysis. Front. Hum. Neurosci..

[13] Corrivetti F, de Schotten MT, Poisson I, Froelich S, Descoteaux M, Rheault F, Mandonnet E (2019). Dissociating motor-speech from lexico-semantic systems in the left frontal lobe: Insight from a series of 17 awake intraoperative mappings in glioma patients. Brain Struct. Funct..

[14] Zaca D, Jarso S, Pillai J (2013). Role of semantic paradigms for optimization of language mapping in clinical FMRI studies. AJNR Am. J. Neuroradiol..

[15] Li Q, Ferraro GD, Pasquini L, Peck KK, Makse HA, Holodny AI (2020). Core language brain network for FMRI language task used in clinical applications. Netw. Neurosci..

[16] Wong, B., Yin, B. & O’Brien, B. Neurolinguistics: Structure, function, and connectivity in the bilingual brain. *Biomed. Res. Int.* **2016** (2016). 10.1155/2016/7069274 PMC4736376 26881224

[17] Li Q, Dong JW, Del Ferraro G, Petrovich Brennan N, Peck KK, Tabar V, Makse HA, Holodny AI (2019). Functional translocation of broca’s area in a low-grade left frontal glioma: Graph theory reveals the novel, adaptive network connectivity. Front. Neurol..

[18] Dorogovtsev SN, Goltsev AV, Mendes JFF (2006). K-core organization of complex networks. Phys. Rev. Lett..

[19] Bullmore E, Sporns O (2009). Complex brain networks: Graph theoretical analysis of structural and functional systems. Nat. Rev. Neurosci..

[20] Hermundstad AM, Bassett DS, Brown KS, Aminoff EM, Clewett D, Freeman S, Frithsen A, Johnson A, Tipper CM, Miller MB (2013). Structural foundations of resting-state and task-based functional connectivity in the human brain. Proc. Natl. Acad. Sci. USA.

[21] Gallos LK, Makse HA, Sigman M (2012). A small world of weak ties provides optimal global integration of self-similar modules in functional brain networks. Proc. Natl. Acad. Sci. USA.

[22] Klimidis S, Reddy P, Minas IH, Lewis J (2004). Brief functional english proficiency measure for health survey research. Aust. Psychol..

[23] Marian V, Blumenfeld HK, Kaushanskaya M (2007). The language experience and proficiency questionnaire (leap-q): Assessing language profiles in bilinguals and multilinguals. J. Speech Lang. Hear. Res..

[24] Cox RW (1996). Afni: Software for analysis and visualization of functional magnetic resonance neuroimages. Comput. Biomed. Res..

[25] Benjamin CF, Walshaw PD, Hale K, Gaillard WD, Baxter LC, Berl MM, Polczynska M, Noble S, Alkawadri R, Hirsch LJ (2017). Presurgical language FMRI: Mapping of six critical regions. Hum. Brain Mapp..

[26] Mandonnet E (2017). A surgical approach to the anatomo-functional structure of language. Neurochirurgie.

[27] Matsuo K, Kato C, Sumiyoshi C, Toma K, Thuy DHD, Moriya T, Fukuyama H, Nakai T (2003). Discrimination of exner’s area and the frontal eye field in humans-functional magnetic resonance imaging during language and saccade tasks. Neurosci. Lett..

[28] Sarubbo S, Tate M, De Benedictis A, Merler S, Moritz-Gasser S, Herbet G, Duffau H (2020). Mapping critical cortical hubs and white matter pathways by direct electrical stimulation: An original functional atlas of the human brain. Neuroimage.

[29] Fedorenko E, Kanwisher N (2009). Neuroimaging of language: Why hasn’t a clearer picture emerged?. Lang. Linguist. Compass.

[30] Peck KK, Bradbury M, Petrovich N, Hou BL, Ishill N, Brennan C, Tabar V, Holodny AI (2009). Presurgical evaluation of language using functional magnetic resonance imaging in brain tumor patients with previous surgery. Neurosurgery.

[31] Kriegeskorte N, Simmons WK, Bellgowan PS, Baker CI (2009). Circular analysis in systems neuroscience: The dangers of double dipping. Nat. Neurosci..

[32] Bastian, M., Heymann, S., & Jacomy, M. Gephi: An Open Source Software for Exploring and Manipulating Networks (2009).

[33] Isaacs KL, Barr WB, Nelson PK, Devinsky O (2006). Degree of handedness and cerebral dominance. Neurology.

[34] Xia M, Wang J, He Y (2013). Brainnet viewer: A network visualization tool for human brain connectomics. PLoS ONE.

[35] Carlson NR (2012). Physiology of Behavior.

[36] Burleson-Lesser K, Morone F, Tomassone MS, Makse HA (2020). K-core robustness in ecological and financial networks. Sci. Rep..

[37] Roux F-E, Draper L, Köpke B, Démonet J-F (2010). Who actually read exner? Returning to the source of the frontal “writing center” hypothesis. Cortex.

[38] Mariën P, Ackermann H, Adamaszek M, Barwood CH, Beaton A, Desmond J, De Witte E, Fawcett AJ, Hertrich I, Küper M (2014). Consensus paper: Language and the cerebellum: An ongoing enigma. Cerebellum.

[39] Cho NS, Peck KK, Zhang Z, Holodny AI (2018). Paradoxical activation in the cerebellum during language FMRI in patients with brain tumors: Possible explanations based on neurovascular uncoupling and functional reorganization. Cerebellum.

[40] Honey C, Sporns O, Cammoun L, Gigandet X, Thiran J-P, Meuli R, Hagmann P (2009). Predicting human resting-state functional connectivity from structural connectivity. Proc. Natl. Acad. Sci. USA.

[41] Seo R, Stocco A, Prat CS (2018). The bilingual language network: Differential involvement of anterior cingulate, basal ganglia and prefrontal cortex in preparation, monitoring, and execution. Neuroimage.

[42] Li L, Emmorey K, Feng X, Lu C, Ding G (2016). Functional connectivity reveals which language the control regions “control” during bilingual production. Front Hum Neurosci.

[43] Rubinov M, Sporns O (2010). Complex network measures of brain connectivity: Uses and interpretations. Neuroimage.

[44] Morone F, Makse HA (2015). Influence maximization in complex networks through optimal percolation. Nature.

[45] Catani M, Dell’Acqua F, Vergani F, Malik F, Hodge H, Roy P, Valabregue R, De Schotten MT (2012). Short frontal lobe connections of the human brain. Cortex.

[46] Friederici AD (2011). The brain basis of language processing: From structure to function. Physiol. Rev..

[47] Luk G, Bialystok E, Craik FI, Grady CL (2011). Lifelong bilingualism maintains white matter integrity in older adults. J. Neurosci..

[48] Olulade OA, Seydell-Greenwald A, Chambers CE, Turkeltaub PE, Dromerick AW, Berl MM, Gaillard WD, Newport EL (2020). The neural basis of language development: Changes in lateralization over age. Proc. Natl. Acad. Sci. USA.

[49] Black MM, Walker SP, Fernald LC, Andersen CT, DiGirolamo AM, Lu C, McCoy DC, Fink G, Shawar YR, Shiffman J (2017). Early childhood development coming of age: Science through the life course. Lancet.

[50] Basho S, Palmer ED, Rubio MA, Wulfeck B, Müller R-A (2007). Effects of generation mode in FMRI adaptations of semantic fluency: Paced production and overt speech. Neuropsychologia.

[51] Darkow R, Martin A, Würtz A, Flöel A, Meinzer M (2017). Transcranial direct current stimulation effects on neural processing in post-stroke aphasia. Hum. Brain Mapp..

[52] Meinzer M, Flaisch T, Breitenstein C, Wienbruch C, Elbert T, Rockstroh B (2008). Functional re-recruitment of dysfunctional brain areas predicts language recovery in chronic aphasia. Neuroimage.

