# Supplementary material for: Monolingual and bilingual language networks in healthy subjects using functional MRI and graph theory
Source: Sci Rep. 2021 May 19;11:10568. doi: 10.1038/s41598-021-90151-4 (PMC8134560; doi:10.1038/s41598-021-90151-4)
Supplement: Supplementary file 1 — Supplementary Information 1. [file 41598_2021_90151_MOESM1_ESM.pdf]

Monolingual and bilingual language networks in healthy subjects using functional MRI and graph theory

Qiongge Li[1,2,3*†], Luca Pasquini[4,5†], Gino Del Ferraro[1,4,6], Madeleine Gene[4], Kyung K. Peck[4,7], Hernán A. Makse[1], Andrei I. Holodny[4,8,9]

[1]Levich Institute and Physics Department, City College of New York, New York, NY 10031, USA
[2]Department of Physics, Graduate Center of City University of New York, New York, NY 10016, USA
[3]Department of Radiation oncology and molecular radiation sciences, Johns Hopkins University School of Medicine, Baltimore, MD 21205, USA
[4]Department of Radiology, Memorial Sloan Kettering Cancer Center, New York, NY 10065, USA
[5]Neuroradiology Unit, NESMOS Department, Sant'Andrea Hospital, La Sapienza University, Rome, RM 00189, Italy
[6]Center for Neural Science, New York University, New York, NY 10003, USA
[7]Department of Medical Physics, Memorial Sloan Kettering Cancer Center, New York, NY 10065, USA
[8]New York University School of Medicine, New York, NY 10016, USA
[9]Department of Neuroscience, Weill Medical College of Cornell University, New York, NY 10065, USA

September 20, 2020

* Corresponding author, Email: qli78@jhmi.edu
† Equal contribution

Supplementary Information

1. K-SHELL DECOMPOSITION ALGORITHM EXPLANATION

Starting from the whole network (1-core), as in **a)**, we begin by disconnecting all nodes with degree equal to 1 ($k = 1$) and then recalculate the degrees for each node left in the network and continue to remove nodes with an updated degree of 1, as shown in **b)**. The nodes disconnected during this step are called nodes in 1-shell (k-shell, where $k = 1$) and the remaining graph makes up 2-core ($(k+1)$-core, where $k = 1$) as in **c)**. Next, we increase k to 2 and repeat this pruning process until the whole network is disassembled. The final removed nodes are in the 3-shell (k_{max} shell) and the final subnetwork just before collapse is called the 3-core(k_{max}-core).

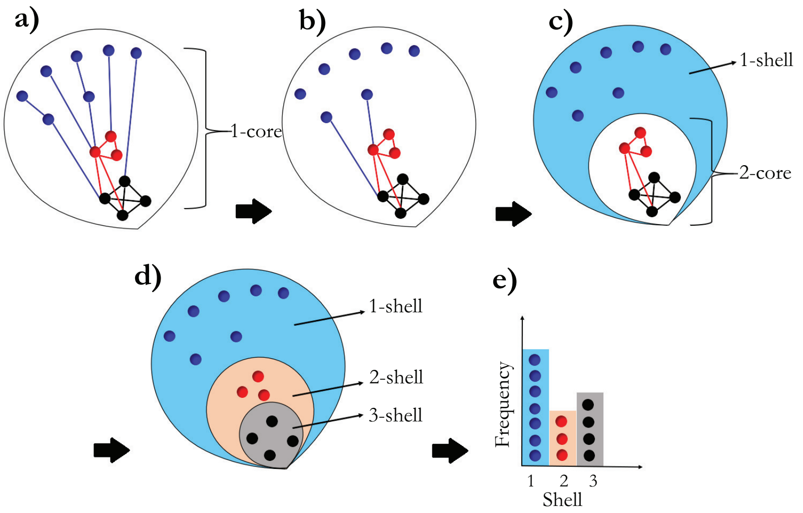

Supplementary Figure S1: **Schematic representation of a network undergoing k-shell decomposition**. In step **a)**, the network begins from 1-core (or $k = 1$), which comprises the whole network. In step **b)**, all nodes with a degree equal to 1 are disconnected. After updating the degrees, there are two more nodes with a degree equal to 1. We continue to disconnect these nodes. In step **c)**, we have disconnected all nodes with a degree equal to 1 and there are no more nodes with a degree less than 2. The remaining graph comprises the 2-core (or $k = 2$; the nodes are still connected). These nodes, which were removed from both **a)** and **b)** composed the 1-shell. The 1-shell and 2-core are exclusive from one another. We continue this process (here omitting $k = 2$ and 3) until step **d)**, where all nodes are disconnected; here, k reaches its maximum, which is 3. Step **e)** is to plot a k-shell histogram where the horizontal axis represents the shell number and the vertical axis represents the counts in each shell.

2. ACTIVE AREAS

Supplementary Table S1: Active areas across subjects.

Activated areas	Abbreviations	Activated in # of monolingual subjects	Activated in # of bilingual English-speaking subjects	Activated in # of bilingual Spanish-speaking subjects
Angular Gyrus(L)	AngG(L)	12.5%	50.0%	37.5%
Broca's Area(L)	BA(L)	100.0%	100.0%	100.0%
Broca's Area(R)	BA(R)	25.0%	0.0%	0.0%
Caudate(L)	Caudate(L)	12.5%	12.5%	25.0%
Caudate(R)	Caudate(R)	0.0%	0.0%	12.5%
Deep Opercular Cortex(L)	DOC(L)	25.0%	37.5%	50.0%
Deep Opercular Cortex(R)	DOC(R)	25.0%	0.0%	0.0%
anterior-Middle Frontal Gyrus(L)	a-MFG(L)	50.0%	75.0%	75.0%
anterior-Middle Frontal Gyrus(R)	a-MFG(R)	25.0%	12.5%	37.5%
ventral-Premotor Area(L)	v-preMA(L)	100.0%	100.0%	100.0%
dorsal-Premotor Area(L)	d-preMA(L)	62.5%	50.0%	50.0%
Premotor Area(R)	preMA(R)	12.5%	0.0%	0.0%
pre-Supplementary Motor Area	pre-SMA	100.0%	100.0%	100.0%
Supra-Marginal Gyrus(L)	SupraMG(L)	62.5%	50.0%	62.5%
Supra-Marginal Gyrus(R)	SupraMG(R)	25.0%	0.0%	0.0%
Wernicke's Area(L)	WA(L)	75.0%	50.0%	100.0%
Wernicke's Area(R)	WA(R)	50.0%	12.5%	12.5%

3. LINK WEIGHTS IN INDIVIDUAL NETWORKS

Supplementary Table S2: Link weight in monolinguals

Link label	fROI pairs #/Subjects	1	2	3	4	5	6	7	8
A	BA(L) - v-preMA(L)	3.94	3.27	8.11	1.96	0.01	1.13	3.03	4.10
B	pre-SMA - v-preMA(L)	5.25	1.44	2.80	3.03	0.89	0.43	1.39	1.26
C	pre-SMA - BA(L)	1.99	0.88	2.07	2.43	0.26	0.12	0.09	0.40
D	BA(L) - WA(L)	0.00	0.19	1.09	1.75	0.00	0.01	0.01	0.02
E	WA(L) - v-preMA(L)	0.00	0.93	0.01	1.03	0.00	0.05	0.01	0.16

Supplementary Table S3: Link weight in bilingual English-speakers

Link label	fROI pairs #/Subjects	1	2	3	4	5	6	7	8
A	BA(L) - v-preMA(L)	1.24	1.25	4.27	2.68	2.81	1.79	1.60	1.93
B	pre-SMA - v-preMA(L)	4.24	0.01	4.88	1.64	1.78	0.04	2.70	1.58
C	pre-SMA - BA(L)	1.49	0.14	0.74	0.89	1.64	0.18	0.01	0.77
D	BA(L) - WA(L)	0.00	0.00	0.00	0.29	0.06	0.04	0.00	0.05
E	WA(L) - v-preMA(L)	0.00	0.00	0.00	0.27	0.01	0.09	0.00	0.03

Supplementary Table S4: Link weight in bilingual Spanish-speakers

Link label	fROI pairs #/Subjects	1	2	3	4	5	6	7	8
A	BA(L) - v-preMA(L)	8.29	1.59	11.46	1.05	4.32	0.60	2.91	2.56
B	pre-SMA - v-preMA(L)	6.61	0.75	5.21	2.58	5.62	1.05	2.18	4.11
C	pre-SMA - BA(L)	2.68	0.82	1.53	0.89	3.54	0.05	0.29	1.97
D	BA(L) - WA(L)	0.02	0.17	0.01	0.67	0.11	0.01	0.02	0.02
E	WA(L) - v-preMA(L)	0.05	0.18	0.42	0.01	0.01	0.08	0.18	0.06

4. LINK WEIGHTS IN COMMON NETWORKS

Supplementary Table S5: Link weight in monolingual common network

Link label	fROI pairs	mean $\pm$ stdv
A	BA(L) - v-preMA(L)	3.20 $\pm$ 2.28
B	pre-SMA - v-preMA(L)	2.06 $\pm$ 1.46
C	pre-SMA - BA(L)	1.03 $\pm$ 0.91
D	BA(L) - WA(L)	0.51 $\pm$ 0.67
E	WA(L) - v-preMA(L)	0.37 $\pm$ 0.44

Supplementary Table S6: Link weight in bilingual English-speaker common network

Link label	fROI pairs #/Subjects	mean $\pm$ stdv
A	BA(L) - v-preMA(L)	2.19 $\pm$ 0.96
B	pre-SMA - v-preMA(L)	2.11 $\pm$ 1.65
C	pre-SMA - BA(L)	0.73 $\pm$ 0.57
D	BA(L) - WA(L)	0.11 $\pm$ 0.11
E	WA(L) - v-preMA(L)	0.10 $\pm$ 0.10

Supplementary Table S7: Link weight in bilingual Spanish-speaker common network

Link label	fROI pairs #/Subjects	mean $\pm$ stdv
A	BA(L) - v-preMA(L)	4.10 $\pm$ 3.60
B	pre-SMA - v-preMA(L)	3.51 $\pm$ 2.05
C	pre-SMA - BA(L)	1.47 $\pm$ 1.13
D	BA(L) - WA(L)	0.13 $\pm$ 0.21
E	WA(L) - v-preMA(L)	0.13 $\pm$ 0.13
